# Transbronchoscopic Local Injection of Triamcinolone Acetonide for Subglottic Granulation Tissue: A Case Report

**DOI:** 10.1002/rcr2.70556

**Published:** 2026-04-05

**Authors:** Keigo Sudo, Kentaro Minegishi, Takaya Sato, Masaya Sogabe, Shunsuke Endo, Hiroyoshi Tsubochi

**Affiliations:** ^1^ Department of Thoracic Surgery Saitama Medical Center Jichi Medical University Saitama Japan

**Keywords:** subglottic granulation tissue, transbronchoscopic local injection, triamcinolone acetonide

## Abstract

Subglottic granulation tissue is a rare but recurrent cause of airway stenosis that develops after epithelial injury from endotracheal intubation or other insults. We herein report a 19‐year‐old woman who developed progressive dyspnoea 2 months after intubation for autoimmune encephalitis. Bronchoscopy revealed a pedunculated subglottic mass, which was resected with an electrocautery snare. Histology confirmed granulation tissue. Despite initial therapy, recurrence occurred within 2 weeks. A second bronchoscopic resection was performed, followed by the local injection of 8 mg of triamcinolone acetonide (TA) into the base of the lesion. Subsequent bronchoscopic surveillance over 4 months demonstrated no recurrence. TA exerts anti‐inflammatory and antifibrotic effects and, when administered locally, it provides a high drug concentration with minimal systemic toxicity. Only a few cases of airway stenosis due to granulation tissue treated transbronchoscopic TA injection have been reported. This minimally invasive approach is a safe and effective treatment option for recurrent airway granulation lesions.

## Graphical abstract

This case report describes the treatment of a recurrent subglottic granulation tissue using bronchoscopic triamcinolone acetonide (TA) injection. This minimally invasive approach is a safe and effective treatment option for recurrent airway granulation lesions.

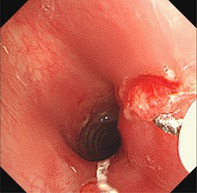

## 1 Introduction

Granulation tissue formation in the airway can occur following epithelial injury caused by infection, trauma, endotracheal intubation, tracheostomy, or tracheal stent placement [1]. Subglottic granulation tissue is an uncommon but potentially recurrent cause of airway stenosis that can be challenging to manage. We herein report a case of **s**ubglottic granulation tissue following endotracheal intubation, successfully treated with local triamcinolone acetonide (TA) injection using flexible bronchoscopy.

## 2 Case Report

A 19‐year‐old woman with no prior medical history underwent emergency endotracheal intubation with a 7.5‐mm internal diameter tube for post‐infectious autoimmune encephalitis. She was extubated after 12 days, following clinical improvement with systemic steroid therapy, and was discharged 1 month later.

**FIGURE 1:**
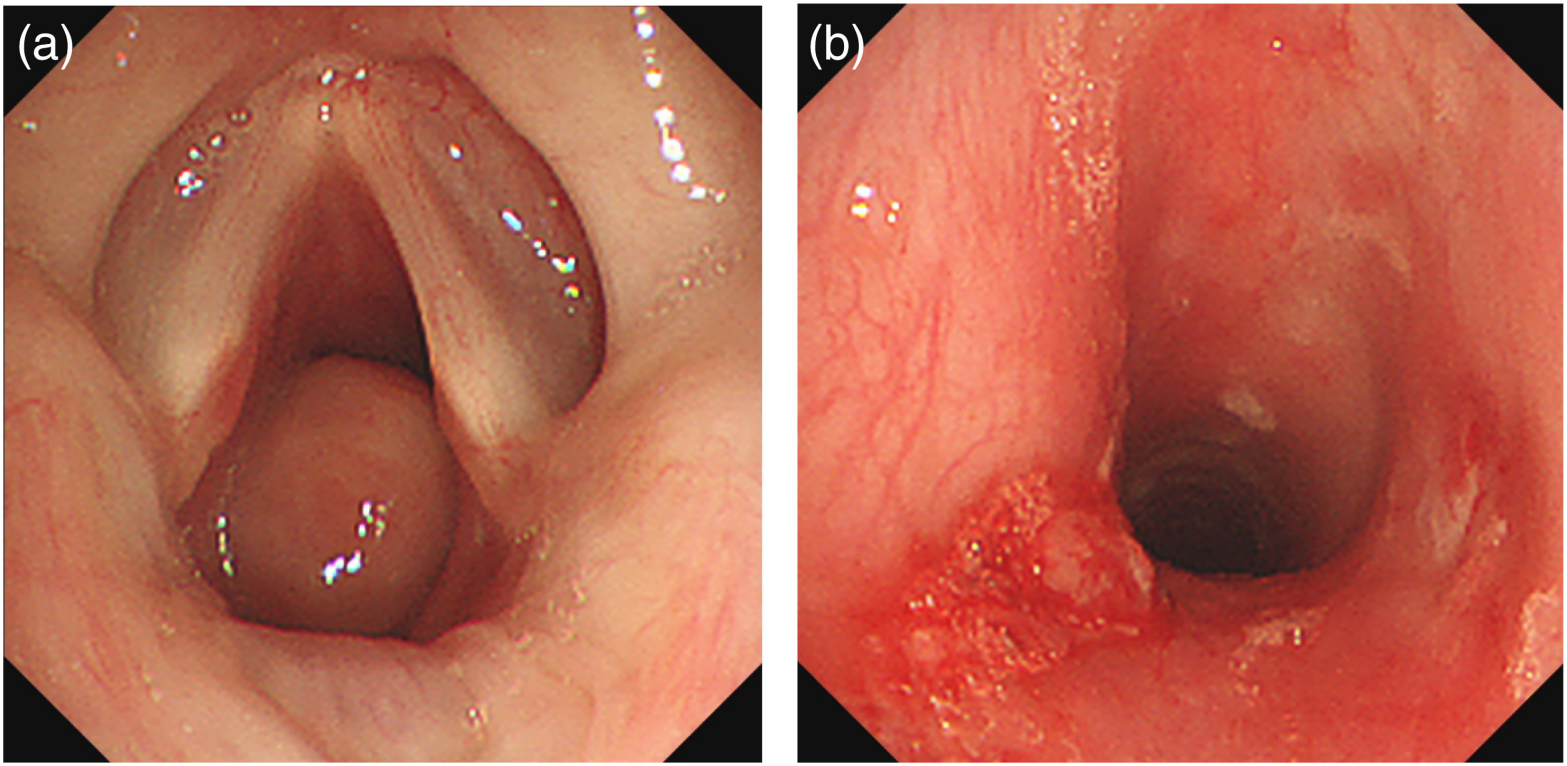
(a) Bronchoscopy revealed a tumour located below the glottis. (b) The tumour was cauterised with a snare and resected using flexible bronchoscope.

Two months after extubation, she developed progressive dyspnoea. Bronchoscopy revealed a pedunculated mass in the subglottic area causing airway narrowing (Figure 1a). The lesion, which had two stalks that arose from the membranous portion of the trachea, was resected using an electrocautery snare using flexible bronchoscopy (Figure 1b). Histopathological examination confirmed granulation tissue, consistent with post‐intubation subglottic granulation tissue. Postoperatively, antiallergic agents and inhaled corticosteroids were administered to prevent recurrence.

**FIGURE 2:**
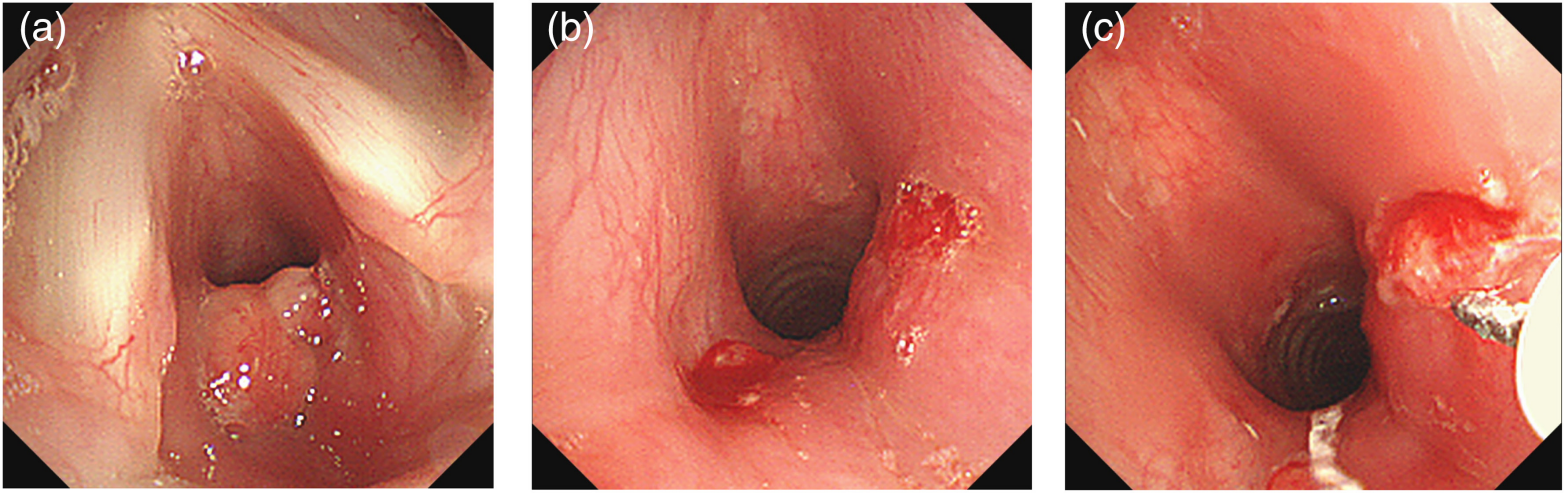
(a) Bronchoscopy demonstrated recurrence of the subglottic tumour. (b) The tumour was cauterised with a snare and resected again. (c) Triamcinolone acetonide was injected transbronchoscopically using an endoscopic injection needle.

**FIGURE 3:**
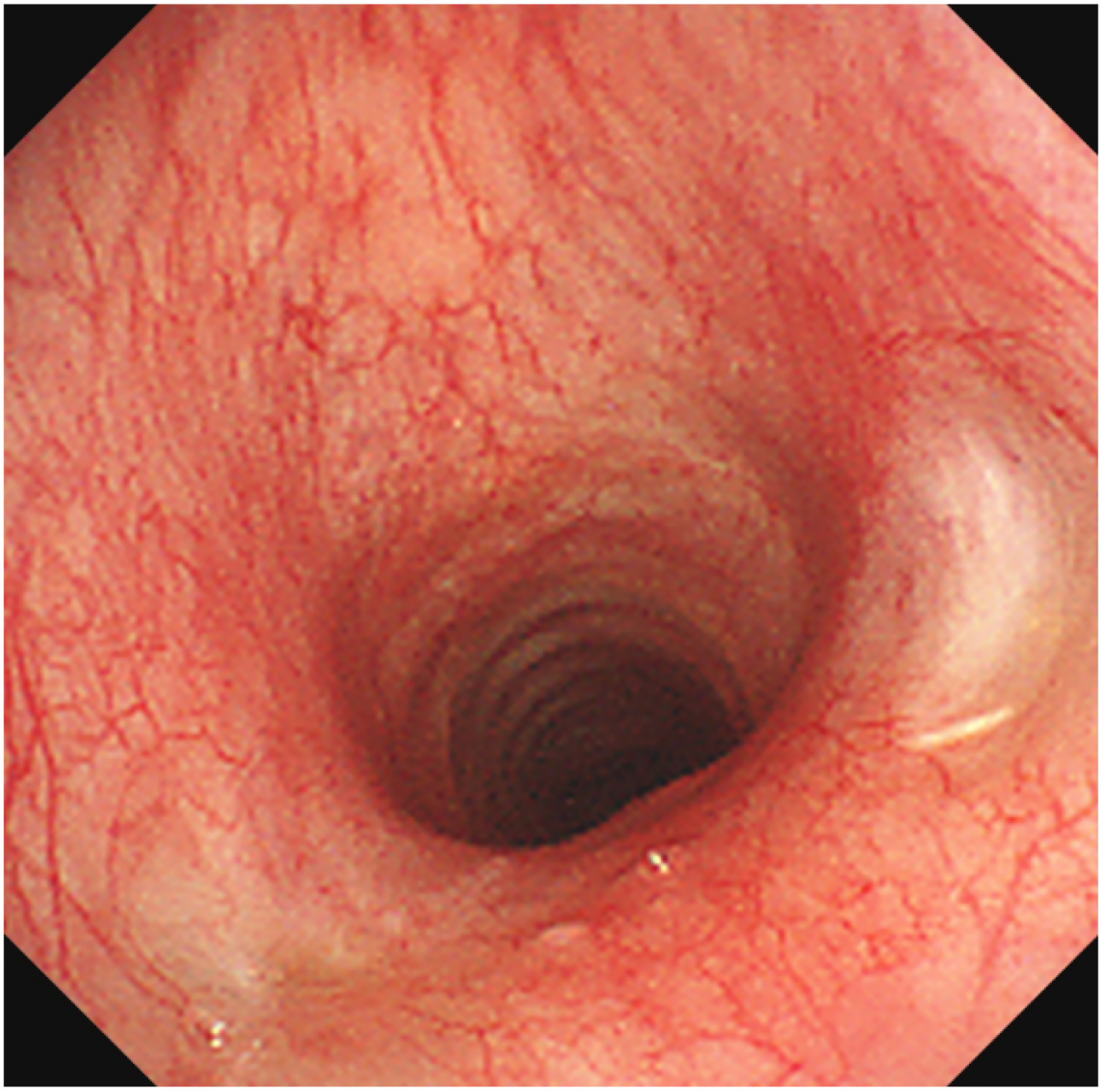
Follow‐up bronchoscopy showed no evidence of recurrence 4 months after resection.

However, 2 weeks later, surveillance bronchoscopy revealed a recurrent subglottic granulation tissue (Figure 2a). Following excision of the recurrent granulation tissue, local injection of TA was planned. The lesion was resected again using an electrocautery snare (Figure 2b). Immediately following resection, a total of 8 mg (0.2 mL) of TA was locally injected into the base of lesion using an endoscopic injection needle (25‐gauge, 3‐mm needle length, short bevel, 2600‐mm total length; Clear Flow, Top Co. Ltd., Tokyo, Japan) (Figure 2c). Follow‐up bronchoscopies were performed every 2–3 weeks for 4 months, confirming the absence of recurrence (Figure 3). The patient is currently under follow‐up observation.

## 3 Discussion

Airway granulation develops when epithelial injury leads to persistent inflammation, ulceration, and exposure of the submucosa and perichondrium. Prolonged inflammation may prevent mucosal regeneration, promoting granulation tissue formation [1]. In post‐intubation subglottic granulation tissue, proposed mechanisms include tracheal chondritis due to mechanical trauma, secondary infection, or chronic irritation [2]. Notably, such lesions have been reported even after a single short‐duration intubation performed solely for surgery [3], and the minimum duration required for onset remains unclear.

Treatment options for airway stenosis vary depending on etiology and location, and include surgical resection, stenting, and endoscopic interventions such as local ethanol injection or topical mitomycin C. In otolaryngology, benign laryngeal granulation tissues, including subglottic granulation tissue, have been treated with laser ablation combined with local TA injection or steroid inhalation. Local TA injection has been reported as an effective and safe treatment for vocal nodules and refractory vocal process granulation tissue via percutaneous or laryngeal endoscopic approaches, as well as through tracheostomy for subglottic granulation tissue [4]. To date, only a few cases have been reported of granulation‐induced tracheal or bronchial stenosis being treated with TA injection via flexible bronchoscopy [5, 6]. This approach offers a less invasive option for subglottic granulation tissue, which is difficult to access with a conventional laryngoscope.

While no standardised dosage of TA exists, previous reports have used single doses of 5–16 mg (less than 1 mg/kg) [4]. TA exerts anti‐inflammatory effects, inhibits vascular endothelial growth factor, and suppresses fibroblast proliferation and collagen synthesis. It may also promote collagen degradation through collagenase activation and inhibition of collagenase antagonists, and disrupt abnormal collagen cross‐links via glucocorticoid receptor‐mediated mechanisms. Local steroid injection allows a high concentration at the lesion site with minimal systemic adverse effects. As a long‐acting synthetic corticosteroid, TA provides prolonged therapeutic effects, favourable absorption, and a low risk of allergic reaction [7].

Transbronchoscopic local injection of TA is regarded as a minimally invasive and highly effective therapeutic approach for subglottic granulation tissue. In particular, we consider this approach to be useful for the management of recurrent cases.

## Author Contributions

Kentaro Minegishi gave final approval of the version to be published. All authors read and approved the final manuscript.

## Funding

The authors have nothing to report.

## Consent

The authors declare that written informed consent was obtained for the publication of this manuscript and accompanying images and attest that the form used to obtain consent from the patient complies with the Journal requirements as outlined in the author guidelines.

## Conflicts of Interest

The authors declare no conflicts of interest.

## Data Availability

The data that support the findings of this study are available on request from the corresponding author. The data are not publicly available due to privacy or ethical restrictions.

## References

[1] J. Gavilan , M. A. Cerdeira , and A. Toledano , “Surgical Treatment of Laryngotracheal Stenosis: A Review of 60 Cases,” Annals of Otology, Rhinology, and Laryngology 107, no. 7 (1998): 588–592, 10.1177/000348949810700708. 9682854

[2] R. T. Barton , “Observation on the Pathogenesis of Laryngeal Granuloma due to Endotracheal Anesthesia,” New England Journal of Medicine 248 (1953): 1097–1099, 10.1056/NEJM195306252482604. 13054897

[3] G. Cuestas , V. Rodriguez , F. Doormann , P. B. Munzon , and G. B. Munzon , “Post‐Intubation Laryngeal Granuloma: A Rare Complication of Tracheal Intubation in Pediatrics. Case Report,” Archivos Argentinos de Pediatría 115, no. 5 (2017): e315–e318, 10.5546/aap.2017.e315. 28895711

[4] S. H. Lee , J. O. Yeo , J. I. Choi , et al., “Local Steroid Injection via the Cricothyroid Membrane in Patients With a Vocal Nodule,” Archives of Otolaryngology – Head & Neck Surgery 137, no. 10 (2011): 1011–1016, 10.1001/archoto.2011.168. 22006779

[5] R. Izumi , Y. Moroe , and R. Kato , “Three Cases of Stenosis Caused by Granulation Successfully Treated by Bronchoscopic Intralesional Injection of Triamcinolone Acetonide,” Journal of the Japan Society for Bronchology 21 (1999): 358–364.

[6] T. Niwa , A. Nakamura , T. Kato , et al., “Bronchoscopic Intralesional Injection of Triamcinolone Acetonide Treated Against Bronchial Obstruction Caused by Peanut Aspiration,” Respiratory Medicine 99, no. 5 (2005): 645–647, 10.1016/j.rmed.2004.10.002. 15823464

[7] T. M. Holder , K. W. Ashcraft , and L. Leape , “The Treatment of Patients With Esophageal Strictures by Local Steroid Injections,” Journal of Pediatric Surgery 4, no. 6 (1969): 646–653, 10.1016/0022-3468(69)90492-8. 5371094

